# Effect of different administration times of dexmedetomidine on the ED50 of sufentanil to inhibit the cardiovascular response in elderly patients with double lumen tracheal intubation: a randomized controlled trial

**DOI:** 10.1186/s12871-026-03747-6

**Published:** 2026-04-28

**Authors:** Bin Lu, Ying Wang, Chen Wang, Lin Wang, Tiantian Wang, Hui Lin, Zhihua Wang

**Affiliations:** 1 Department of Anesthesiology, Hainan Affiliated Hospital of Hainan Medical University (Hainan General Hospital), Haikou, 570311 China; 2 Department of Anesthesiology, Guangdong Provincial Hospital of Chinese Medicine Hainan Hospital, Haikou, 570203 China

**Keywords:** Sufentanil, Dexmedetomidine, Double-lumen tracheal intubation, Dixon up-and-down method, Median effective dose

## Abstract

**Objective:**

To investigate the effect of different administration timings of dexmedetomidine (DEX) on the median effective dose (ED50) of sufentanil required to suppress the cardiovascular response during double-lumen tracheal intubation in elderly patients.

**Methods:**

Ninety elderly patients (aged 65–85 years, BMI 18.5–30 kg/m^2^, ASA physical status I–III) scheduled for elective thoracic surgery were randomized into three groups: the Control group (induction initiated following a 10-min intravenous infusion of saline), the DEX1 group (DEX 0.6 μg/kg infused over 10 min before induction), and the DEX2 group (induction initiated 20 min after completion of a 10-min intravenous infusion of DEX at a dose of 0.6 μg/kg). Anesthesia induction was initiated with intravenous sufentanil starting at 0.5 μg/kg, and subsequent doses were determined using the Dixon up-and-down sequential method with a dose ratio of 1:1.1 between adjacent dose levels. Tracheal intubation was performed 5 min after sufentanil administration according to the predefined protocol, and the infusion-to-intubation interval was predefined according to group allocation. Sequential allocation continued until at least eight crossover pairs were obtained. Patient characteristics, hemodynamic variables, Surgical Pleth Index (SPI), and adverse events were recorded from study drug administration to 5 min after intubation.

**Results:**

Final analysis included 87 patients (Control: *n =* 30, DEX1: *n =* 28, DEX2: *n =* 29). The ED50 of sufentanil was 0.675 μg/kg (95% CI: 0.634–0.732 μg/kg) in the Control group, 0.507 μg/kg (95% CI: 0.474–0.540 μg/kg) in the DEX1 group, and 0.396 μg/kg (95% CI: 0.330–0.440 μg/kg) in the DEX2 group, corresponding to relative reductions of 24.9% and 41.3%, respectively.

**Conclusion:**

Premedication with dexmedetomidine reduced the ED50 of sufentanil required to suppress the cardiovascular response to double-lumen tracheal intubation in elderly patients, with earlier administration producing a greater opioid-sparing effect. Administration of DEX 30 min before anesthesia induction resulted in the lowest sufentanil requirement.

**Trial registration:**

This study was registered at the China Clinical Trial Registration Center (ChiCTR2400083508, April 26, 2024).

**Supplementary Information:**

The online version contains supplementary material available at 10.1186/s12871-026-03747-6.

## Background

The global incidence of lung cancer in elderly patients is steadily increasing [1], driving a corresponding rise in geriatric thoracic surgeries. Double-lumen endotracheal tubes (DLTs) are a cornerstone in thoracic surgery, enabling effective one-lung ventilation and optimizing surgical conditions. However, the intubation process using DLTs is inherently more complex due to their unique design, which introduces significant mechanical and nociceptive stimulation [2]. This stimulation frequently triggers robust cardiovascular responses, especially in elderly patients, whose autonomic nervous system is less adaptable. The hemodynamic fluctuations may subsequently increase the incidence of postoperative cardiovascular and cerebrovascular complications [3], necessitating a careful balance in perioperative management.

To suppress this cardiovascular stress response, high doses of opioid such as sufentanil are often required, but this can lead to significant hemodynamic instability in elderly patients, including risks of hypotension, bradycardia, and delayed recovery. Consequently, there is a pressing need for adjunctive agents that can enhance cardiovascular stability while minimizing opioid requirements. Dexmedetomidine (DEX), a selective α−2 adrenergic receptor agonist, is a promising option for sedation, anxiolysis, and analgesia, effectively suppressing sympathetic outflow. DEX, with an onset of 10–15 min and peak effects at 30 min [4, 5], is increasingly utilized as an adjuvant in general anesthesia induction, especially in vulnerable elderly populations.

Although previous studies have demonstrated the efficacy of DEX in improving perioperative hemodynamic stability and reducing opioid requirements, critical gaps remain. The optimal timing of DEX administration relative to intubation and its impact on the dose–response relationship with sufentanil remain poorly understood. This study seeks to establish the median effective dose (ED50) of sufentanil required to suppress cardiovascular responses during DLT intubation when used with DEX. Additionally, it seeks to evaluate how different administration timings of DEX influence the ED50 of sufentanil, providing evidence-based insights to optimize anesthetic protocols for elderly patients who have undergone thoracic surgery.

## Methods

### Trial registration

This study was conducted in accordance with the Declaration of Helsinki, was approved by the Institutional Review Board of Hainan Provincial People's Hospital (Approval ID: 2023–427) and registered at http://www.chictr.org.cn, China Clinical Trial Registration Center (Registration Number: ChiCTR2400083508) on April 26, 2024. This randomized controlled trial was designed, conducted, and reported in accordance with the CONSORT 2010 statement. The first patient was enrolled on April 30, 2024, confirming that trial registration was completed prospectively prior to participant enrollment.

### Patients and study design

All participants provided written informed consent prior to inclusion and received a detailed explanation of the study’s purposes and procedures. Between April 30 and June 30, 2024, 90 patients aged 65–85 years with a BMI of 18.5–30 kg/m^2^ and ASA physical status I–III, scheduled for thoracic surgery with double-lumen endotracheal tube intubation under general anesthesia, were enrolled in the study at Hainan Provincial People's Hospital.

Patients with any of the following conditions were excluded:

- Allergy to anesthetic drugs.
- History of sedative drug abuse or recent opioid use.
- Poorly controlled hypertension (baseline systolic blood pressure [SBP] ≥ 160 mmHg upon admission).
- Arrhythmias, encompassing atrial fibrillation, frequent premature ventricular contractions; tachycardia (HR > 100 beats/min); bradycardia (HR < 50 beats/min).
- History of myocardial infarction or cerebral infarction.
- Liver or kidney insufficiency.
- Cormack and Lehane (CL) grade ≥ 3.
- Repeated adjustment of the double-lumen bronchial tube positioning or requiring > 2 intubation attempts.
- Severe hypotension (if mean arterial pressure [MAP] of the patient was ≤ 60 mmHg) after administration of study drugs, requiring treatment with vasoactive drugs.

### Randomization and blinding

The SPSS 26.0 software was utilized to generate a random number scale, with each random number corresponding to a specific group. An anesthesiologist who did not participate in the data collection or analysis of this study placed the card indicating the group into an opaque envelope, which was then sealed within a light-opaque box. After the patient entered the operating room, this anesthesiologist drew an envelope from the box and prepared the study drug according to the group card contained within it. Allocation concealment was ensured using sequentially numbered opaque sealed envelopes. Blinding was implemented for patients and statisticians. The anesthesiologist performing tracheal intubation, the investigator responsible for cardiovascular response assessment, and the investigator responsible for assigning subsequent sufentanil doses in the sequential allocation process were all blinded to group allocation.

Patients were randomised to one of the following groups:

- *Control group*: Induction was initiated following a 10-min intravenous infusion of saline.
- *DEX1 group*: Induction was initiated following the administration of a 10-min intravenous infusion of dexmedetomidine, with a dosage of 0.6 μg/kg.
- *DEX2 group*: Induction was initiated 20 min after completing a 10-min intravenous infusion of dexmedetomidine, with a dosage of 0.6 μg/kg.

### Outcomes

The primary outcome of this study was the median effective dose (ED50) of sufentanil required to suppress the cardiovascular response to double-lumen tracheal intubation. Secondary outcomes included hemodynamic variables (mean arterial pressure [MAP] and heart rate [HR]), Surgical Pleth Index (SPI), and adverse events (hypotension and bradycardia).

### Surgical procedure and clinical observations

Patients fasted for 8 h and abstained from drinking for 6 h prior to surgery. Venous access was established through the dorsal hand vein of the patient's left upper limb upon entering the operating room. Sodium lactate Ringer's solution was administered intravenously at 18 mL·kg⁻1·h⁻1 for preoperative volume expansion, with a total of 10 mL/kg infused [6]. Invasive arterial puncture monitoring was conducted.

Baseline measurements of mean arterial pressure (MAP), heart rate (HR), and oxygen saturation (SpO₂) were recorded upon admission to the operating room (T0). The baseline value was defined as the stabilized physiological value recorded at T0, prior to administration of the study drug, and this definition was prespecified to provide a uniform physiological reference across groups before dexmedetomidine administration.

A positive cardiovascular response was defined as an increase in MAP or HR of ≥ 20% from baseline, assessed using the predefined fixed post-intubation measurement time points (T3 and T4). If either time point met the criterion, the response was considered positive.

### Study drug administration

The study drug was prepared and administered by an anesthetist nurse who was not involved in data recording or analysis, and was subsequently handed over to an anesthesiologist who also did not participate in data recording or analysis. Patients received the study drug before surgery, and 2 L/min of pure oxygen was administered during the infusion period. Hemodynamic parameters (MAP and HR) and oxygen saturation (SpO₂) were recorded immediately before anesthesia induction (T1). In the Control and DEX1 groups, T1 coincided with completion of the 10-min study drug infusion, whereas in the DEX2 group, T1 was recorded 20 min after completion of the dexmedetomidine infusion according to the predefined protocol.

### Anesthesia induction and intubation procedure

Anesthesia induction was then initiated according to the study protocol: immediately after completion of the infusion in the Control and DEX1 groups, and 20 min after completion of the dexmedetomidine infusion in the DEX2 group. Induction was initiated with intravenous administration of sufentanil over 1 min, followed by slow injection of 0.3 mg/kg etomidate. Once adequate sedation was achieved (Observer’s Assessment of Alertness/Sedation [OAA/S] score ≤ 3), 0.3 mg/kg of cis-atracurium was administered intravenously. Hemodynamic variables (MAP, HR, and SpO₂) were recorded 5 min after the administration of sufentanil (T2).

Laryngoscopy was performed 5 min after the start of sufentanil administration, once adequate neuromuscular relaxation was achieved. The elapsed time from the start and completion of dexmedetomidine infusion to tracheal intubation was recorded for all patients. According to the predefined protocol, the interval from the start of study drug infusion to tracheal intubation was approximately 15 min in the Control and DEX1 groups and approximately 35 min in the DEX2 group. A double-lumen endotracheal tube was then inserted using a video laryngoscope (TD-C-IV Zhejiang Youyi Medical Device Co., Ltd.) by an anesthesiologist with more than 5 years of thoracic anesthesia experience. Tube size was selected based on the patient’s left main bronchial diameter, height, and weight [7].

The double-lumen endotracheal intubation was completed within 60 s. Intubation was considered successful if no further adjustment of tube position or repeated intubation was required. After successful intubation, both lungs were ventilated using a mechanical ventilator. Hemodynamic variables (MAP, HR) and SpO₂ were recorded immediately after intubation (T3) and at 5 min after intubation (T4), with T4 defined as the fixed measurement time point 5 min after tracheal intubation [8].

**Fig. 1:**
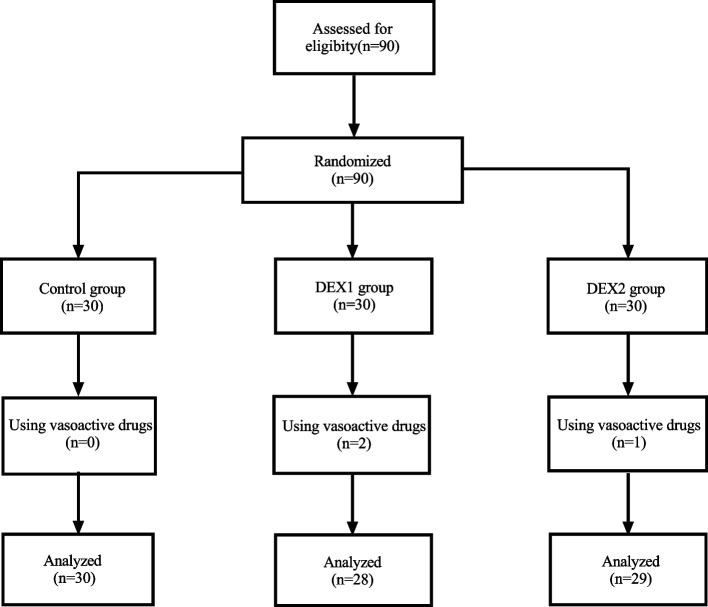
Patient selection flowchart illustrates the sequence of selection in the Control group, DEX1, and DEX2

The Surgical Pleth Index (SPI) was monitored only at T2, T3, and T4, as SPI is less correlated with pain stimulation in conscious patients. The study protocol is illustrated in Fig. 1.

### Adverse events and rescue interventions

Adverse reactions immediately after intubation and within 5 min of intubation were recorded. These included:

- Hypotension: MAP ≤ 60 mmHg [9].
- Bradycardia: HR < 50 beats/minute [10].
- Severe hypotension: MAP ≤ 55 mmHg [11].
- Severe bradycardia: HR ≤ 45 beats/min.

During the infusion of the study drug, anesthesia induction, and endotracheal intubation, the following interventions were administered if necessary:

- Severe hypotension (MAP ≤ 55 mmHg): 0.1–0.3 mg of metaraminol.
- Severe bradycardia (HR ≤ 45 beats/min): 0.4–0.5 mg of atropine.
- Severe hypotension and bradycardia: 1–2 mg of dopamine.

Patients requiring rescue vasoactive medication were excluded from the sequential ED50 allocation analysis because the up-and-down design requires a definitive binary response for dose assignment; however, these patients were excluded only from the ED50 sequential analysis and were retained in safety reporting.

### Median effective dose measurement

The sufentanil dosage was determined using the Dixon up-and-down sequential allocation method. Based on preliminary study findings, the initial sufentanil dose was set at 0.50 μg/kg. Subsequent doses were adjusted according to the Dixon up-and-down method using a dose ratio of 1:1.1 between adjacent dose levels; if a positive cardiovascular response occurred, the dose administered to the next patient was increased by one dose level, whereas a negative response resulted in a decrease by one dose level. Accordingly, the dose ladder included 0.50, 0.55, 0.61, 0.67, and 0.75 μg/kg, with additional higher or lower doses generated according to the same ratio as required by the sequential allocation process. All doses were rounded to two decimal places for clinical preparation. Sequential allocation continued until at least eight crossover pairs (response reversals) were observed in each group, consistent with standard Dixon up-and-down methodology.

### Determination of sample size

The Dixon up-and-down approach suggests that the recommended number of subjects per group is 20–40 in study design [12]. Researchers typically account for potential participant exclusions by estimating a 10% attrition rate during planning phases. Based on these considerations, the study protocol specifies enrolling 30 participants per study cohort (*n =* 30) to accommodate possible dropouts. Data collection continued until at least eight response reversals (crossovers) were observed, after which enrollment for that group was terminated.

### Statistical analyses

Statistical analyses were performed using SPSS software (version 26.0). To assess the normality of continuous variables, the Shapiro–Wilk test was used. Variables following a normal distribution were presented as mean ± standard deviation (x̅ ± s), whereas non-normally distributed variables were shown as median (M). Categorical variables were summarized as frequencies (n) or percentages (%).

The median effective dose (ED50) and corresponding 95% confidence intervals were estimated separately for each group using probit regression analysis. Because the up-and-down sequential design is primarily intended for ED50 estimation rather than formal between-group hypothesis testing, comparisons of ED50 differences between groups were interpreted descriptively using percentage differences and confidence interval overlap rather than inferential hypothesis testing.

Where applicable, statistical significance was considered at the *p* < 0.05 level. Sequential line charts were created using GraphPad Prism software (version 8).

## Results

A total of 90 patients were randomized. Three patients (two in the DEX1 group and one in the DEX2 group) required rescue vasoactive intervention before response assessment. Because a definitive binary response (success/failure) is required for sequential dose allocation, these cases could not contribute to the ED50 estimation process. Therefore, ED50 was estimated based on 87 patients (30 in the Control group, 28 in the DEX1 group, and 29 in the DEX2 group). All 90 randomized patients were included in safety analyses (Fig. 1). No statistically significant differences were observed among the three groups in terms of age, sex, height, weight, BMI, ASA grade, CL grade, intubation time, or tracheal tube size (Table 1).

**Table 1:** Characteristics of study groups

	Control group (*n =* *30*)	DEX1 group (*n =* *28*)	DEX2 group (*n =* *29*)	*P*
Age (y)	70 ± 4.0	72 ± 4.0	71 ± 5.0	0.143
Male/Female (n)	17/13	18/10	20/9	0.613
Height (cm)	161 ± 8.3	163 ± 7.6	160 ± 8.0	0.453
Weight (kg)	55.2 ± 7.7	58.3 ± 8.7	58.9 ± 10.4	0.259
BMI (kg/m^2^)	21.4 ± 2.8	21.9 ± 2.4	22.7 ± 3.0	0.186
ASA I/II/III	0/29/1	0/26/2	0/27/2	0.781
Time for intubation (s)	45 ± 3	47 ± 5	46 ± 3	0.313
CL grade I/II	16/14	17/11	15/14	0.768
Type Tube (Fr) 32/35/37	12/11/7	9/13/6	8/17/4	0.562
Type Laryngoscope 4/5	13/17	8/20	9/20	0.444

Control group: Induction was initiated following a 10-min intravenous infusion of saline; DEX1 group: induction was initiated following a 10-min intravenous infusion of dexmedetomidine at a dose of 0.6 μg/kg; DEX2 group: induction was initiated 20 min after completing a 10-min intravenous infusion of dexmedetomidine at a dose of 0.6 μg/kg

Data conforming to a normal distribution were reported as mean ± standard deviation (x̅ ± s), while non-normally distributed variables were expressed as median (M). Categorical variables were summarized as frequencies (n)

Type of laryngoscope (4/5) refers to Macintosh blade size 4 or 5

*BMI* Body mass index, *CL grade* Cormack and Lehane grade

**Fig. 2:**
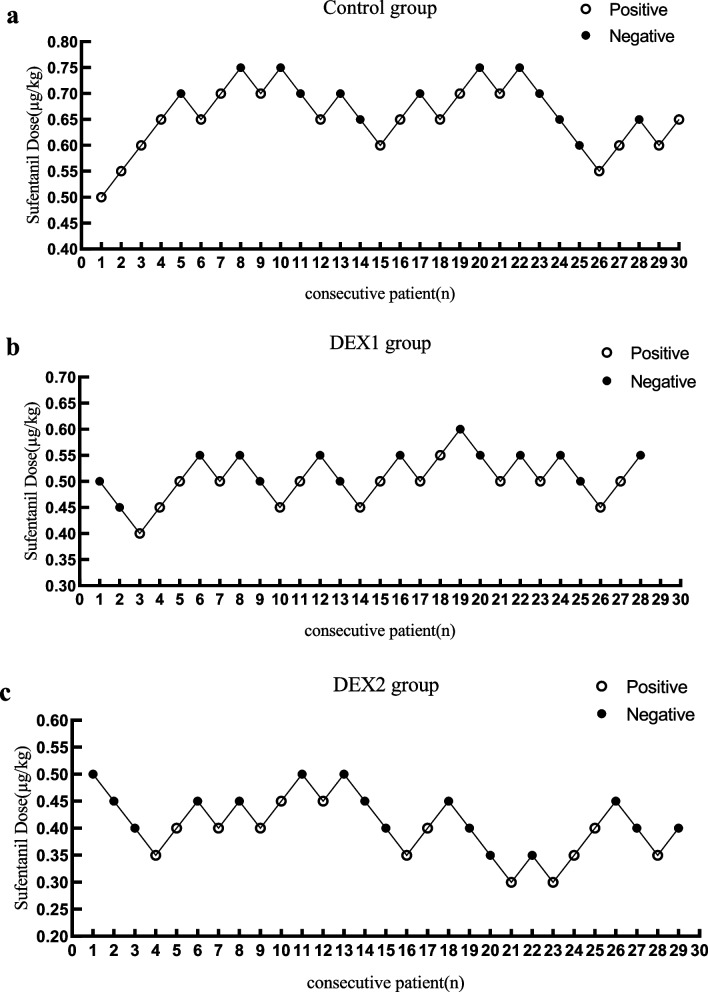
Sequential allocation of cardiovascular responses to intubation in three groups using the Dixon up-and-down method. **a** Control group; **b** DEX1 group; **c** DEX2 group. The starting dose in each group was 0.5 μg/kg, and the dose interval between consecutive patients followed a 1:1.1 ratio. Open circles indicate positive responses, and filled circles indicate negative responses

ED50 estimates are presented descriptively with corresponding 95% confidence intervals. The estimated ED50 of sufentanil was lower in the DEX1 group (0.507 μg/kg, 95% CI 0.474–0.540 μg/kg) and in the DEX2 group (0.396 μg/kg, 95% CI 0.330–0.440 μg/kg) than in the Control group (0.675 μg/kg, 95% CI 0.634–0.732 μg/kg). Compared with the Control group, the ED50 of sufentanil was reduced by 24.9% in the DEX1 group and by 41.3% in the DEX2 group, suggesting a potential timing-related effect of dexmedetomidine on reducing sufentanil requirements for inhibiting cardiovascular responses during double-lumen tracheal intubation (Fig. 2).

### Hemodynamic and SPI profiles during intubation

Hemodynamic data at each time point are presented descriptively in (Table 2).

**Table 2:** MAP in different groups and time points

Time	Control group (*n =* 30)	DEX1 group (*n =* 28)	DEX2 group (*n =* 29)
T_0_	101.4 ± 7.7	100.0 ± 8.8	100.7 ± 8.9
T_1_	97.4 ± 7.4	90.1 ± 9.7	84.4 ± 10.7
T_2_	76.4 ± 9.6	76.5 ± 11.1	72.7 ± 10.9
T_3_	122.5 ± 19.4	111.9 ± 16.5	110.4 ± 17.7
T_4_	103.7 ± 14.5	88.4 ± 17.4	96.3 ± 17.4

Control group: Induction was initiated following a 10-min intravenous infusion of saline; DEX1 group: induction was initiated following a 10-min intravenous infusion of dexmedetomidine at a dose of 0.6 μg/kg; DEX2 group: induction was initiated 20 min after completing a 10-min intravenous infusion of dexmedetomidine at a dose of 0.6 μg/kg

*MAP* Mean arterial pressure, *T*_*0*_ the time point of entering the operating room, *T*_*1*_ immediately before anesthesia induction, *T*_*2*_ the time point of 5 min after the administration of sufentanil, *T*_*3*_ the time point of immediately post-intubation, *T*_*4*_ the time point 5 min after intubation

Within-group temporal changes in MAP were observed following intubation.

Because sufentanil doses differed systematically across groups by design of the sequential allocation method, formal between-group statistical comparisons of secondary hemodynamic outcomes were not emphasized. At T1, lower HR values were observed in the DEX2 group compared with the Control group, whereas HR values in the DEX1 group appeared similar to those in the Control group at this time point (Table 3).

**Table 3:** HR in different groups and time points

Time	Control group (*n =* 30)	DEX1 group (*n =* 28)	DEX2 group (*n =* 29)
T_0_	72.6 ± 9.1	76.1 ± 8.3	73.1 ± 9.5
T_1_	70.2 ± 9.5	67.4 ± 9.5	62.4 ± 8.0
T_2_	61.0 ± 7.1	60.1 ± 8.9	57.6 ± 8.2
T_3_	88.6 ± 11.7	87.4 ± 13.0	81.0 ± 12.4
T_4_	73.7 ± 13.4	67.6 ± 12.0	69.4 ± 12.7

T_0_: the time point of entering the operating room; T_1_: immediately before anesthesia induction; T_2_: the time point of 5 min after the administration of sufentanil; T_3_: the time point of immediately post-intubation; T_4_: the time point 5 min after intubation

*HR* Heart rate

The Surgical Pleth Index (SPI) showed distinct temporal patterns across groups. At T2, SPI values appeared lower in the DEX2 group relative to both the DEX1 and Control groups. At T3 and T4, lower SPI values were observed in both dexmedetomidine groups compared with the Control group. At T4, SPI values in the DEX1 and DEX2 groups were comparable to each other (Table 4).

**Table 4:** Different group and time-point comparisons of SPI

Time	Control group (*n =* 30)	DEX1 group (*n =* 28)	DEX2 group (*n =* 29)
T_2_	35.2 ± 12.3	26.8 ± 10.0	19.3 ± 9.3
T_3_	66.5 ± 12.7	56.9 ± 18.0	56.0 ± 13.1
T_4_	52.4 ± 16.4	36.6 ± 16.7	36.1 ± 16.8

T_2_: the time point of 5 min after the administration of sufentanil; T_3_: the time point of immediately post-intubation; T_4_: the time point 5 min after intubation; SPI: surgical pleth index

These descriptive patterns suggest potential modulation of nociceptive responses during intubation in patients receiving dexmedetomidine. However, given the systematic differences in sufentanil exposure inherent to the sequential allocation design, these observations should be interpreted as exploratory.

No apparent differences were observed in the incidence of bradycardia or hypotension across the three groups (Table 5).

**Table 5:** Adverse events

	Control group (*n =* 30)	DEX1 group (*n =* 28)	DEX2 group (*n =* 29)
Bradycardia	0 (0)	1 (4)	1 (3)
Hypotension	1 (3)	2 (7)	5 (17)

Categorical variables were summarized as frequencies (n) or percentages (%)

Values represent the lowest recorded MAP and HR between T1 and T4 after study drug administration

The distribution of administered sufentanil doses differed across groups in accordance with the sequential allocation design (Supplementary Table S1).

## Discussion

This study demonstrates that premedication with dexmedetomidine reduced the median effective dose (ED50) of sufentanil required to suppress cardiovascular responses during double-lumen tracheal intubation in elderly patients. The effect appeared to be time-dependent, with administration 30 min before induction producing a greater reduction in sufentanil requirements. These findings highlight the importance of optimizing the timing of dexmedetomidine administration to improve hemodynamic stability.

More specifically, administration of dexmedetomidine 30 min before anesthesia induction (DEX2 group) resulted in an ED50 of 0.396 μg/kg (95% CI 0.330–0.440 μg/kg), compared with 0.507 μg/kg (95% CI 0.474–0.540 μg/kg) in the DEX1 group and 0.675 μg/kg (95% CI 0.634–0.732 μg/kg) in the Control group. This corresponded to reductions of 41.3% and 24.9%, respectively, suggesting that earlier administration of dexmedetomidine produced greater opioid-sparing effects.

Clinically, double-lumen endotracheal tube intubation is a common method for one-lung ventilation in thoracic surgery [13, 14]. However, its unique curvilinear design increases the risk of significant hemodynamic fluctuations during intubation [13]. These fluctuations are primarily attributed to reflex sympathetic excitation, which elevates blood pressure, heart rate, and catecholamine release [15]. Elderly patients are particularly vulnerable to exaggerated cardiovascular responses due to age-related increases in norepinephrine levels and heightened autonomic nervous system activity [16]. Although opioids like sufentanil are widely recognized for their effectiveness in mitigating nociceptive responses during intubation and ensuring perioperative analgesia [17–19], their administration in elderly patients poses challenges. High doses of sufentanil can provoke dramatic hemodynamic changes, highlighting the critical need to optimize its dosage during double-lumen endobronchial intubation in this population.

### Mechanisms underlying dexmedetomidine’s effects

The reduction in sufentanil requirements observed with dexmedetomidine administration is likely attributable to activation of presynaptic α2-adrenergic receptors in the central nervous system and vascular endothelium, which suppresses central sympathetic outflow and reduces norepinephrine release both centrally and peripherally, thereby contributing to hemodynamic modulation [20, 21]. Previous studies have demonstrated that the timing and dose of dexmedetomidine administration are important determinants of its cardiovascular effects. For example, Scheinin et al. [22] administered a loading dose of dexmedetomidine 0.6 μg/kg infused over 10 min before laryngoscopy, which attenuated cardiovascular responses to intubation in healthy adults. Similarly, other studies reported that dexmedetomidine administered prior to anesthesia induction at doses ranging from 0.5 to 1 μg/kg reduced perioperative opioid requirements and showed trends toward improved peri-intubation hemodynamic control [23–25].

In the present study, synchronization of dexmedetomidine peak plasma concentration with tracheal intubation likely explains the superior results observed in the DEX2 group, as this approach allows maximal attenuation of the stress response. The selection of an appropriate loading dose is particularly important in elderly patients to balance efficacy and safety. Previous evidence suggests that loading doses ≥ 0.7 μg/kg are associated with an increased incidence of hypotension, especially in elderly individuals ^[21]^, whereas adequate sedation (Ramsay sedation score ≥ 3) has been achieved with an ED95 of approximately 0.86 μg/kg [26]. Considering these findings, a loading dose of 0.6 μg/kg administered before anesthesia induction was selected in the present study to optimize both safety and clinical efficacy.

### Hemodynamic and nociceptive modulation

In this sequential dose-finding study, descriptive hemodynamic patterns were observed across groups during the peri-intubation period. Differences in MAP and HR trajectories were noted at specific time points; however, because sufentanil doses differed systematically across groups by design of the up-and-down allocation method, these observations likely reflect the combined effects of dexmedetomidine timing and variable opioid exposure.

Accordingly, secondary hemodynamic and SPI findings should be interpreted as exploratory and hypothesis-generating rather than confirmatory. The study was not designed or powered for formal between-group comparisons of secondary outcomes, and no superiority inference can be made regarding timing strategies.

Nevertheless, the observed temporal patterns are pharmacologically plausible. Dexmedetomidine exerts sympatholytic effects through presynaptic α₂-adrenergic receptor activation, which may contribute to modulation of stress responses during intubation. Future adequately powered parallel-group trials with fixed opioid dosing would be required to determine whether specific timing strategies confer clinically meaningful hemodynamic advantages.

### Clinical implications

These findings underscore the importance of timing dexmedetomidine administration to coincide with peak stress periods during intubation. Administering dexmedetomidine 30 min before induction appears to provide optimal cardiovascular and nociceptive stabilization. This timing-dependent effect emphasizes the need for tailored anesthetic protocols, particularly in elderly patients who are more vulnerable to exaggerated stress responses and adverse drug effects.

### Limitations of this study

Several limitations of this study should be acknowledged. First, only a single dexmedetomidine loading dose (0.6 μg/kg) was evaluated; therefore, the potential effects of alternative dexmedetomidine dosing regimens on the ED50 of sufentanil were not explored. Future studies investigating multiple dexmedetomidine dosing strategies in combination with different opioid induction regimens are warranted to better define optimal dosing approaches.

Second, to ensure consistency across groups, the baseline reference used for determining cardiovascular responses was defined as the stabilized pre-drug physiological value recorded at T0. However, the use of a pre-drug baseline rather than a pre-intubation reference value may influence interpretation of cardiovascular responses, and this methodological aspect should be further examined in future studies.

Third, elderly patients with significant cardiovascular, cerebrovascular, hepatic, or renal diseases were excluded, which may limit the generalizability of the present findings to broader elderly surgical populations. Future studies including patients with common comorbidities are needed to better evaluate the clinical applicability of dexmedetomidine in real-world elderly surgical settings.

In addition, patients who required rescue vasoactive medication were excluded from the sequential ED50 allocation analysis because the up-and-down method requires a definitive binary response outcome for dose assignment; however, these patients were retained in safety reporting. Consequently, a strict intention-to-treat analysis was not fully feasible, and this approach may introduce potential selection bias that should be considered when interpreting the findings. This analytical approach deviates from a conventional intention-to-treat framework but reflects a methodological characteristic inherent to sequential dose-finding designs, in which a definitive binary response is required for dose allocation.

Finally, the sequential up-and-down design, while appropriate for ED50 estimation, required a relatively small sample size and did not allow comprehensive evaluation of perioperative hemodynamic variability, opioid consumption, or adverse events. Because this design is optimized for ED50 estimation, reliable estimation of higher quantiles such as ED95 was not feasible given the sample size requirements of this method. Furthermore, the study was designed to estimate the ED50 within each group rather than to detect statistically powered between-group differences; therefore, comparisons between groups should be interpreted descriptively. Larger prospective trials using parallel dose–response designs are warranted to enable formal between-group comparisons and estimation of clinically relevant endpoints such as ED95.

## Conclusion

In summary, the ED50 of sufentanil required to inhibit the cardiovascular response to double-lumen endotracheal intubation in elderly patients was 0.507 μg/kg when dexmedetomidine (0.6 μg/kg) was infused 10 min before anesthesia induction and 0.396 μg/kg when infused 30 min before induction. Administration of dexmedetomidine 30 min before anesthesia induction resulted in the lowest sufentanil requirement. This study provides valuable insights into the optimal timing and dosing of dexmedetomidine to minimize sufentanil requirements.

## Supplementary Information

Supplementary Material 1: Supplementary Table S1. Distribution of administered sufentanil doses during the sequential allocation process by study group.

Supplementary Material 2.

## Data Availability

The datasets used and/or analyzed during the current study are available from the corresponding author on reasonable request.

## Abbreviations

DEX: Dexmedetomidine
ED50: Median effective dose
DLT: Double-lumen tracheal tube
MAP: Mean arterial pressure
HR: Heart rate
SPI: Surgical Pleth Index
OAA/S: Observer’s Assessment of Alertness/Sedation
