# Supplementary material for: Effect of different administration times of dexmedetomidine on the ED50 of sufentanil to inhibit the cardiovascular response in elderly patients with double lumen tracheal intubation: a randomized controlled trial
Source: BMC Anesthesiol. 2026 Apr 28;26:267. doi: 10.1186/s12871-026-03747-6 (PMC13123098; doi:10.1186/s12871-026-03747-6)
Supplement: Supplementary file 1 — Supplementary Material 1: Supplementary Table S1. Distribution of administered sufentanil doses during the sequential allocation process by study group. [file 12871_2026_3747_MOESM1_ESM.docx]

Supplementary Table S1. Distribution of administered sufentanil doses during the sequential allocation process by study group

| Group | Median dose (μg/kg) | IQR  (μg/kg) | Range  (μg/kg) |
| --- | --- | --- | --- |
| Control | 0.65 | 0.61–0.70 | 0.50–0.75 |
| DEX1 | 0.50 | 0.50–0.55 | 0.40–0.60 |
| DEX2 | 0.40 | 0.35–0.45 | 0.30–0.50 |

*Values are presented as median (interquartile range) and range. Doses represent the actual sufentanil doses administered to participants during the up-and-down sequential allocation process.*
