# Supplementary material for: Effect of different administration times of dexmedetomidine on the ED50 of sufentanil to inhibit the cardiovascular response in elderly patients with double lumen tracheal intubation: a randomized controlled trial
Source: BMC Anesthesiol. 2026 Apr 28;26:267. doi: 10.1186/s12871-026-03747-6 (PMC13123098; doi:10.1186/s12871-026-03747-6)
Supplement: Supplementary file 2 — Supplementary Material 2. [file 12871_2026_3747_MOESM2_ESM.docx]

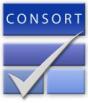


CONSORT 2010 checklist of information to include when reporting a randomised trial_*_

| **Section/Topic** | **Item No** | **Checklist item** | **Reported on page No** |
| --- | --- | --- | --- |
| **Title and abstract** | 1a | Identification as a randomised trial in the title | Page 1 |
|  | 1b | Structured summary of trial design, methods, results, and conclusions _(for_ _specific_ _guidance_ _see_ _CONSORT_ _for_ _abstracts)_ | Page 2-3 |
| **Introduction**  Background and | 2a | Scientific background and explanation of rationale | Page 4 |
| objectives | 2b | Specific objectives or hypotheses | Page 4 |
| **Methods**  Trial design | 3a | Description of trial design (such as parallel, factorial) including allocation ratio | Page 5-6, 9 |
|  | 3b | Important changes to methods after trial commencement (such as eligibility criteria), with reasons | Not applicable |
| Participants | 4a | Eligibility criteria for participants | Page 5–6 |
|  | 4b | Settings and locations where the data were collected | Page 5,7 |
| Interventions | 5 | The interventions for each group with sufficient details to allow replication, including how and when they were | Page 6–8 |
|  |  | actually administered |  |
| Outcomes | 6a | Completely defined pre-specified primary and secondary outcome measures, including how and when they | Page 6–10 |
|  |  | were assessed |  |
|  | 6b | Any changes to trial outcomes after the trial commenced, with reasons | Not applicable |
| Sample size | 7a | How sample size was determined | Page 10–11 |
|  | 7b | When applicable, explanation of any interim analyses and stopping guidelines | Not applicable |
| Randomisation: |  |  |  |
| Sequence | 8a | Method used to generate the random allocation sequence | Page 6 |
| generation | 8b | Type of randomisation; details of any restriction (such as blocking and block size) | Page 6 |
| Allocation | 9 | Page 6 | Page 6 |
| concealment |  | describing any steps taken to conceal the sequence until interventions were assigned |  |
| mechanism |  |  |  |
| Implementation | 10 | Who generated the random allocation sequence, who enrolled participants, and who assigned participants to | Page 6 |
|  |  | interventions |  |
| Blinding | 11a | If done, who was blinded after assignment to interventions (for example, participants, care providers, those | Page 6 |

|  |  | assessing outcomes) and how |  |
| --- | --- | --- | --- |
|  | 11b | If relevant, description of the similarity of interventions | Not applicable |
| Statistical methods | 12a | Statistical methods used to compare groups for primary and secondary outcomes | Page 9–10 |
|  | 12b | Methods for additional analyses, such as subgroup analyses and adjusted analyses | Not applicable |
| **Results**  Participant flow (a | 13a | For each group, the numbers of participants who were randomly assigned, received intended treatment, and | Page 10–11 |
| diagram is strongly |  | were analysed for the primary outcome |  |
| recommended) | 13b | For each group, losses and exclusions after randomisation, together with reasons | Page 11 |
| Recruitment | 14a | Dates defining the periods of recruitment and follow-up | Page 5 |
|  | 14b | Why the trial ended or was stopped | Page 9, 10 |
| Baseline data | 15 | A table showing baseline demographic and clinical characteristics for each group | Page 12 |
| Numbers analysed | 16 | For each group, number of participants (denominator) included in each analysis and whether the analysis was | Page 11 |
|  |  | by original assigned groups |  |
| Outcomes and | 17a | For each primary and secondary outcome, results for each group, and the estimated effect size and its | Page 13–17 |
| estimation |  | precision (such as 95% confidence interval) |  |
|  | 17b | For binary outcomes, presentation of both absolute and relative effect sizes is recommended | Not applicable |
| Ancillary analyses | 18 | Results of any other analyses performed, including subgroup analyses and adjusted analyses, distinguishing | Not applicable |
|  |  | pre-specified from exploratory |  |
| Harms | 19 | All important harms or unintended effects in each group _(for_ _specific_ _guidance_ _see_ _CONSORT_ _for_ _harms)_ | Page 9,16 |
| **Discussion**  Limitations | 20 | Trial limitations, addressing sources of potential bias, imprecision, and, if relevant, multiplicity of analyses | Page 20-21 |
| Generalisability | 21 | Generalisability (external validity, applicability) of the trial findings | Page 21 |
| Interpretation | 22 | Interpretation consistent with results, balancing benefits and harms, and considering other relevant evidence | Page 17-21 |
| **Other information**  Registration | 23 | Registration number and name of trial registry | Page 5 |
| Protocol | 24 | Where the full trial protocol can be accessed, if available | Page 5 |
| Funding | 25 | Sources of funding and other support (such as supply of drugs), role of funders | Page 1 |

*We strongly recommend reading this statement in conjunction with the CONSORT 2010 Explanation and Elaboration for important clarifications on all the items. If relevant, we also recommend reading CONSORT extensions for cluster randomised trials, non-inferiority and equivalence trials, non-pharmacological treatments, herbal interventions, and pragmatic trials. Additional extensions are forthcoming: for those and for up to date references relevant to this checklist, see [www.consort-statement.org.](http://www.consort-statement.org/)
